# A New District Medical Society

**Published:** 1900-05

**Authors:** E. E. Guinn

**Affiliations:** Secretary; Rusk, Texas


### A New District Medical Society.

Rusk, Texas, April 20, 1900.

*Texas Medical Journal, Austin, Texas.*

In compliance with a call issued by the Chairman and Secretary, Drs. Sam R. Burroughs and John H. Evans, of the State Organizing Committee, and indorsed by the local physicians of the city of Palestine, a number of physicians of East Texas met in the above city, in the Comer Club Rooms, at 2 p. m., April 5th, and organized a district medical association, composed of Cherokee, Henderson, Anderson, Houston, Leon and Freestone counties; each county was represented. The attendance was large and enthusiastic. Dr. Sam R. Burroughs, of Buffalo, was made President; Dr. J. H. Evans, of Palestine, First Vice-President; Dr. Wm. C. Lipscomb, of Crockett, Second Vice-President; Dr. E. E. Guinn, of Rusk, Secretary, and Dr. John Colley, of Palestine, Treasurer. Drs. J. R. Milburn, of Rusk, Cherokee county; Dr. Hodges, of Athens, Henderson county; Dr. J. N. Gee, of Bethel, Anderson county; Dr. E. D. Stokes, of Crockett, Houston county; Dr. E. P. Murdock, of Oakwood, Leon county, and Dr. Wm. Sneed, ———, Freestone county, were appointed as a Board of Censors, each county being represented on the board. Dr. A. L. Hathcock, E. M. Link, and J. H. Evans, of Palestine, were appointed on a Committee of Program for the next meeting. The society will meet quarterly, in Palestine, at 2 p. m., on the first Thursdays in April, July, October, and January, and remain in session two days.

There were forty-two names enrolled for membership, and it is earnestly urged that every graduate of a reputable, regular medical college, who is in good standing and residing in this district, shall attend and become a member at the next meeting, on the 5th and 6th day of July. You will be royally entertained. A program of the meeting will appear in this journal in due time. We expect to make this one of the best medical societies in the State.

(Signed) E. E. Guinn, Secretary.

[Keep the ball in motion.—Ed.]

